# Ten simple rules for maximizing summer research experiences for students, mentors, and research groups

**DOI:** 10.1371/journal.pcbi.1013731

**Published:** 2025-11-21

**Authors:** Miriam B. Goodman

**Affiliations:** Department of Molecular and Cellular Physiology, Stanford University, Stanford, California, United States of America; Carnegie Mellon University, UNITED STATES OF AMERICA

## Abstract

Whether hosted by colleges, universities, stand-alone research institutions, federal research labs, or private companies, immersive summer (6–12 weeks) research experiences build students’ confidence in their scientific capabilities and help to refine their professional trajectories. Such internships are an important tool to introduce students to STEM careers and energize participants, each of whom realizes a powerful benefit. The student gains hands-on research experience, insight into the research process, and clarity regarding their educational and career aspirations. The bench mentor, typically an advanced graduate student, postdoctoral researcher, or staff scientist, acquires essential skills in training and mentoring while incorporating fresh perspectives from an inquisitive novice into their research project. The principal investigator (PI) promotes the professional development of the bench mentor, expands interest in STEM careers, while exploring a focused and compact research question. This set of *Ten Simple Rules* is a guide for PIs, bench mentors, and research groups and seeks to foster excellence in the design of short-term research experiences for students. They emphasize projects co-created by PIs and bench mentors, accessible techniques that can be mastered in a few weeks, and strategies enabling interns to develop their own mental model of the research question and approach. Although tailored primarily to full-time summer internships for individual students in an academic research setting, this advice may be applicable to short-term, mentored research experiences in multiple settings.

## Introduction

Summer research experiences are short-term internships offered to undergraduates at colleges, universities, federal research labs, and companies. Whether part of a defined research program or offered on an *ad hoc* basis, they promote a three-way win for scientific research. The first goes to the student intern who gains the chance to do research and to find out how it really works. This is especially meaningful for students who belong to groups historically excluded from research or are unsure about pursuing science for any reason. Consistent with this claim, these experiences help students clarify and refine their desired educational and career path and gain confidence that they can think and work like a scientist [1–3]. The second win goes to the bench mentor who gains supervised experience in training and mentoring others as well as the valuable assistance and perspective of a curious novice. The third goes to the lab head or Principal Investigator (PI) and their research program in the form of branching out to investigate a known unknown that might otherwise go unstudied and in providing focused experience supporting the development of the bench mentor’s skills in the profession. There is an important fourth win, too. Regardless of whether the intern pursues a career in science, their summer experience supports their growth as a learner and helps to build a deeper understanding of research itself, of researchers, and what motivates researchers to make discoveries and to solve problems. This experience may also promote awareness of the value of data-driven and STEM-informed concepts in community projects and public policy.

This set of *Ten Simple Rules* is intended to help mentors make these four wins likely during short-term (8–12 weeks) research experiences. The bench mentor could be an accomplished graduate student, postdoc, laboratory technician, or a staff scientist coached by a group leader, lab head, or PI. In settings like a primarily undergraduate institution (PUI), the bench mentor might be an advanced undergraduate student or a faculty member who is also the lab head [4]. The framework could be adapted to part-time research experiences or to a team of 2–3 interns, supervised by a single bench mentor. Fostering the latter scenario is especially beneficial for bench mentors who are postdocs seeking a future career at a PUI. This variation would provide experience in mentoring multiple students and fostering peer collaboration, strategies that enhance student learning as well as the research capacity and productivity of PUI faculty whose time for research is limited [4]. Such a team-based approach might also be appropriate for summer research experiences outside of academia.

What follows is written primarily for mentors at the bench and at the desk. They are designed to support not only research training but also to welcome all who are curious to the research community and its practices. When recruiting interns, consider that not all potential interns have the financial flexibility to pursue unpaid internships. Consequently, paid opportunities are preferred, and their use makes research experiences accessible to a wider pool of students, broadening participation in science. This framework and workflow are informed by the author’s experience in biomedical research, starting as an intern working writing code in intramural research labs at the National Institutes of Health and continuing to the present day of leading an independent research group at a research-intensive (R1) university for more than two decades. If you are an intern looking for guidance about how to get the most out of your internship, please consult these other “Ten Simple Rules” [5–7].

## Rule 1: A great short-term project is like a great short film

It is focused and specific because there is not enough time to consider more than one question. The plotline is straightforward and uses experimental and analytical techniques that are familiar to the bench mentor and that an intern can master in a period of 1–2 weeks. It is engaging because the project has the potential to generate a novel, if compact discovery. The project’s narrow conceptual and technical focus supports quick and positive feedback, ensuring the intern’s learning, engagement, and development as a scientist.

## Rule 2: The bench mentor and PI co-produce the film

**Fig 1:**
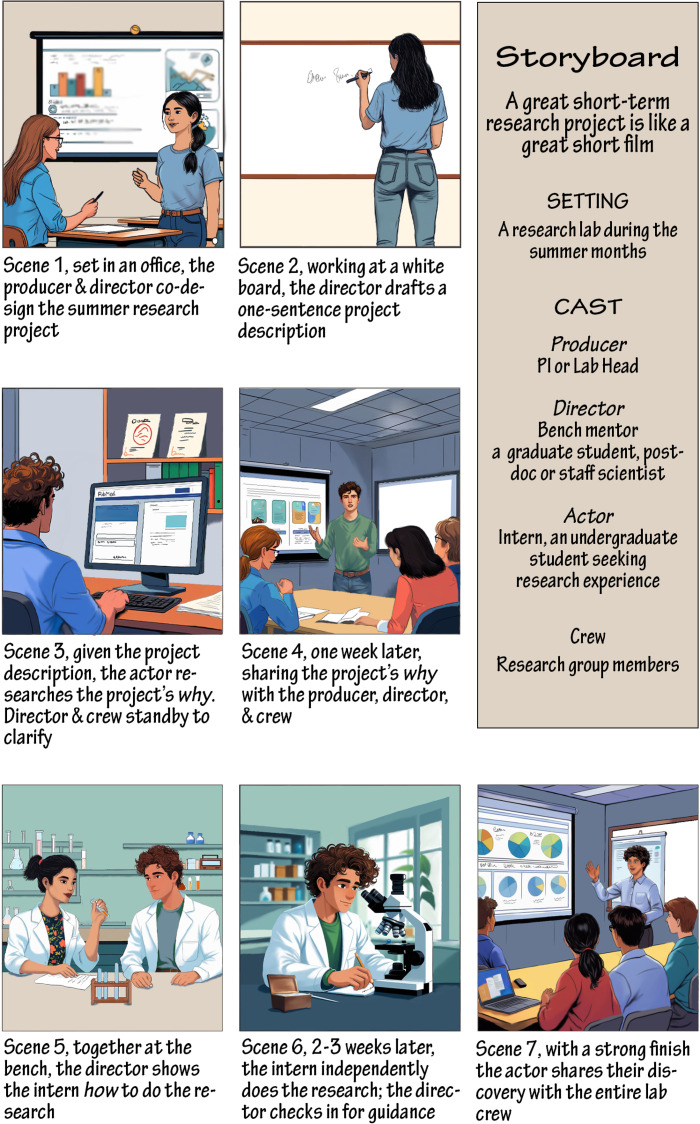
A great summer research experience is like a short film. Storyboard scenes showing some of the events that precede (Scene 1, 2) and make up a short summer research experience (Scene 3–7). The characters in the story are: The Producer, played by the PI or Lab head; The Director, played by the bench mentor; and The Actor, played by the intern. All the members of the research group comprise the crew.

Co-producing and designing the intern’s project refines the scope of the bench mentor’s research project. Effective short-term projects extend the bench mentor’s main research but are tangential to its core goals. The bench mentor’s main research is independent of the intern’s work, creating space for the intern to learn and to make mistakes and new discoveries (Fig 1, Scene 1). Novices bring a fresh perspective to the research project. Consider empowering the intern to focus on validating and characterizing a biological reagent, an emerging technique, or to follow an unexpected observation. Before the project begins, generate a one-sentence description of the project to share with the intern (Fig 1, Scene 2), along with one review and one research article relevant to the project. These papers and their citations are key entry points for self-study by the intern during the first week when they seek to develop an independent understanding of why the research is worth doing (*see* Rule 5).

## Rule 3: The bench mentor directs the film and empowers the intern to act

As the short film’s director, the bench mentor should strive for clarity in communication and for collaboration in research tasks. These actions support quick learning, high-quality experimental work, diligent documentation, and independence. Be explicit. Be welcoming and help the intern acquire an identity as a scientist [8]. Every day is a learning experience, but mastery might require several takes. The ability to identify mistakes and problems as they occur is a central skill to master. One common mistake is for the bench mentor to do the prep work for the intern. This is often demotivating for both the intern and the bench mentor because it can leave the intern unsure of their role and leave the bench mentor feeling like they are working for the intern. In my experience, this outcome can be avoided by ensuring the intern has clarity about which parts of the prep work are their responsibility and which are handled by others. Over time, the intern will become more comfortable seeking guidance and sharing their own ideas about the scientific question being studied.

## Rule 4: The research group is the film’s crew

Every film needs a supporting crew, and the entire research group can fill this need. The bench mentor is responsible for training, supervising, and mentoring the intern, the entire group contributes to positive learning and research outcomes. How? By interacting with the intern and welcoming questions about the discipline, scientific topic, technique, or the safe conduct of research. By modeling the interactive and collaborative nature of research. Some groups ensure that interns meet the entire crew in one-on-one meetings. Others encourage informal connections like inviting an intern to lunch to learn about their goals and uncertainties. Both formal and informal connections with the crew help the intern evaluate the reliability of different sources of information. These interactions also help to reveal the research group’s culture and unwritten rules. For example, the PI or lab head is happy to talk to you, but only if their office door is open. Or, that the lab communicates mostly in person, mostly on email, mostly by messaging, etc. Such practices apply to practical matters as well, such as how to place orders for research supplies, where they are stored, and who to ask for help with specific pieces of equipment.

## Rule 5: Start the research project with the *why* (not the *how*) of the project

Analogous to an actor deciphering their character’s goals and objectives, the first week of the internship is devoted to empowering the intern to develop their own understanding of *why* the project is worth doing and what it might uncover (Fig 1, Scene 3). How? By preparing a short (<15′) presentation and delivering it to the entire research group, who provide feedback (Fig 1, Scene). For most interns, this assignment is neither easy nor familiar. Like a supportive director, the bench mentor should acknowledge its challenges, reinforces its benefits, and encourage the intern to consult multiple sources of information (e.g., bench mentor, lab head, lab crew, online literature, videos, and even generative AI tools). Regardless of the information source, the intern is responsible for the content of their presentation and for understanding the evidence supporting any claims they make. The intern is not expected to work at the bench during this time so that they can spend their time learning how to get up to speed in a new area. This skill is important for doing any kind of research and supports lifelong learning in any domain. Importantly, this assignment helps the intern generate a mental framework for the research project and jumpstarts the engagement of the intern as a thought partner in troubleshooting, analysis, and interpretation.

## Rule 6: The intern is a novice; meet them where they are

Whereas experts can readily distinguish between “I don’t know” and “it is not known,” novices and newcomers struggle to recognize this distinction. In other words, the intern may not know what they don’t know, yet. The intern may also believe, falsely, that the bench mentor and PI expect them to have knowledge specific to the assigned research project before they begin. The bench mentor and the entire lab crew can advance learning by normalizing the fact that everyone starts their research journey as a novice. Be curious about the intern’s knowledge and any gaps in knowledge or skills that you notice. Avoid making assumptions based on their experience or education. Ask the intern “what do you want to know?” and “what seems confusing?” about concepts, techniques, safety, about prior work relevant to the project. Collectively, these conversations create space for learning.

## Rule 7: Teach techniques *stepwise,* starting from the end

Laboratory procedures often involve multiple steps, in which completion depends on a successful start (Fig 1, Scene 5). Novices may struggle to complete procedures because they lack understanding of how the individual steps fit together or which steps are more critical. Consequently, they may be unable to recognize mistakes independently. This impairs decision-making about whether it is better to re-start or forge ahead. Experts like the bench mentor may not be aware of the knowledge they rely on to make such decisions. These human quirks reduce the effectiveness of traditional training methods that involve having the intern watch the bench mentor and then repeat the steps independently from the beginning. Instead, consider a faded demonstration in which the bench mentor performs all steps except the last one, then the last two, then the last three, etc. This strategy is borrowed from research on how people learn and the technique of worked or faded examples in problem solving in STEM classrooms [9,10]. It enables interns to collect data quicker (Fig 1, Scene 6), and this success builds confidence and independence.

## Rule 8: Celebrate mistakes; they are integral to learning

The research process is iterative; skills, knowledge, and insight deepen with each iteration. Mistakes are integral to this process, and novices make more mistakes than experts. Lab heads should ensure that the bench mentor and all crew members give interns space to make mistakes, without compromising safety. Encourage the intern to develop strategies that lessen the chances of making the same mistake twice. Each unique mistake, however, is an opportunity to increase skill, to learn what steps need closer attention, and to develop habits and tools that keep bench research on track. When we recognize and celebrate mistakes, we promote learning, skill development, and, ultimately, create space for more rigorous results.

## Rule 9: Honor and document the process

Integrate record keeping and data management into the internship. Establish naming conventions for digital data files and use them, track metadata associated with each experimental data point. It is common for people to believe they will remember the necessary details; it is rare for people to have perfect recall. Thus, rigorous experimental work requires that the details be documented, including mistakes and missteps. Whether stored on paper or an electronic medium, documentation is a gift to the future and a kindness to our future selves. The same is true of data management. Coach the intern to be kind to themselves, their future selves, and their future collaborators. Let the intern know that these actions would allow the short film to evolve into a full-length feature, carried forward by their bench mentor or other members of the lab crew.

## Rule 10: Finish strong

Empower the intern to share their mastery through a capstone activity, building on the conceptual framework the intern built in week 1. This could be a short written report, a workshop, a poster, or a presentation (Fig 1, Scene 7). Incorporate a reflection of the intellectual distance that the intern traveled during the short-term research project. Did the work address the question posed at the start? If so, what answers were revealed? If not, what got in the way? Help the intern to consider and propose the next steps for growing this research project. Akin to entering a short film in a film festival, encourage the intern to present their research at their home campus, regional conference, or national conference for undergraduate research. Offer to assistance as they advance to their next step. This assistance could take many forms, such as discussions of further training or employment in science, of fellowship funding for future research, or financial support to present their project at a national or international meeting. Keep in contact with your former interns, they are the future of scientific research.

## Conclusion

Much like a great short film, a short-term research project energizes its creators, crew, and audience. Rules 1, 2, and 3 engage the lab head (producer) and bench mentor (director) in co-producing a compact research experience for the intern (actor). During this process, the lab head will coach the bench mentor in “soft” skills, like realistically assessing the scope of a research project and delegating tasks to others in a supportive manner. Rule 4 engages the entire research group (crew). Rules 5, 6, and 7 draw from research into how learning happens and apply them to research training [9,10]. Over the years, I have observed that interns easily immerse themselves in learning *how* to perform research techniques, but find it difficult to grasp *why* their work is worth doing more. Rule 5 deliberately puts the *why* first, empowering the intern to build their own mental model of the research’s goals. Rules 8 and 9 honor the fact that uncertainty is central to research and that experimental failures teach us what we don’t know [11]. Implementing Rule 10 celebrates the intern’s work and reminds the research group that a well-designed and thoughtfully implemented project advances knowledge.

These simple rules can be generalized to other settings and time-bound research experiences. For instance, they could generalize to small teams of interns mentored by a single bench mentor. They might be adapted to an extended research project occurring over the course of a full academic year or spanning multiple years by including the intern in a longer design phase (Rules 1–2). Whether applied to a summer research experience or a variation on this theme, it is my sincere belief that these actions will help you, your research crew, and the intern create a valuable experience marked by: (1) A meaningful research experience for the intern; (2) Improved skills in training, supervising, and strategic decision making in research for the bench mentor; (3) Exploration of an interesting, if tangential, research question for the PI. The experience may help the intern launch a journey to becoming a scientist or clarify their career goals in another direction. Either way, each intern will have mastered something new in weeks and assumed responsibility for evaluating the quality and reliability of sources of information, two skills that generalize to other careers and daily life. Executed with intention and care, summer research experiences for undergraduates fuel curiosity, deepen appreciation for the joy and challenge of STEM research, and open new avenues of scientific discovery for the entire research group.
